# Deep learning-based automated detection of fetal corpus callosum abnormalities in prenatal ultrasound

**DOI:** 10.3389/fped.2026.1774586

**Published:** 2026-04-20

**Authors:** Min Li, Shizhen Liu, Zhonglu Zhang, Qiang Li, Xuan Xu

**Affiliations:** 1 Department of Ultrasonography, Affiliated Hospital of Jining Medical University, Jining, Shandong, China; 2 Department of Rehabilitation Medicine, Jining No.1 People's Hospital, Jining, Shandong, China; 3 School of Data and Computer Science, Shandong Women’s University, Jinan, Shandong, China

**Keywords:** AI-assisted diagnosis, corpus callosum, deep learning, fetal ultrasound, multi-view classification

## Abstract

**Objective:**

Prenatal detection of corpus callosum (CC) abnormalities is essential for assessing fetal neurodevelopment, yet conventional ultrasound diagnosis faces challenges from operator variability and suboptimal fetal positioning.

**Methods:**

We developed a novel deep learning framework CC-FocusNet that integrates automated region localization with an anatomy-aware dual-stream architecture for multi-view analysis. The model was trained on 496 cases and validated on an independent external cohort of 93 cases. We assessed both diagnostic performance and clinical interpretability through attention visualization.

**Results:**

Our framework achieved 97.36% accuracy on the external test set. Grad-CAM++ heatmaps revealed that model attention consistently focused on clinically relevant anatomical landmarks, demonstrating strong interpretability. When integrated into clinical workflows, the AI system enhanced diagnostic accuracy and efficiency, particularly reducing misdiagnosis rates in challenging cases.

**Conclusions:**

This interpretable AI system provides accurate and efficient prenatal detection of CC abnormalities, offering substantial potential to support clinical decision-making and enable timely intervention for at-risk pregnancies.

## 1 Introduction

The corpus callosum (CC), as the major bundle of neural fibers connecting the left and right cerebral hemispheres, plays a central role in the transmission and integration of information between the two halves of the brain [1]. Normal development of the CC is crucial for the structural and functional maturation of the fetal nervous system [2]. Numerous studies have demonstrated that agenesis or dysplasia of the CC is closely associated with a wide range of neurodevelopmental disorders, including intellectual disability, epilepsy, autism spectrum disorder, and cognitive impairment. Therefore, accurate and early detection of CC structure and development during the fetal period is of great clinical value for the early intervention of congenital neurodevelopmental abnormalities.

In current prenatal diagnostic procedures, ultrasonography remains the primary and preferred modality for evaluating the fetal CC [3]. Benefiting from its noninvasive nature, safety, affordability, and real-time imaging capability, ultrasound has become the most widely adopted tool for routine fetal structural screening across medical institutions [4]. Notably, by combining axial and sagittal views for systematic scanning, multimodal ultrasonography provides complementary information on CC morphology, length, and thickness from different anatomical perspectives, thereby offering a solid imaging basis for comprehensive and accurate assessment. In contrast, fetal magnetic resonance imaging (MRI) is typically reserved as a secondary diagnostic tool for resolving equivocal ultrasound findings. Although MRI theoretically provides superior soft-tissue resolution, its high cost, limited accessibility, long acquisition time, and high sensitivity to fetal motion artifacts hinder its use as a routine screening modality. Moreover, MRI interpretation is time-consuming and subjective, relying heavily on the radiologist’s expertise [5]. Consequently, how to fully exploit and utilize the rich and widely available clinical information embedded in multimodal ultrasound data has become a key challenge for improving both the efficiency and accuracy of prenatal diagnosis.

To enhance diagnostic efficiency and accuracy, artificial intelligence (AI) technologies have been increasingly introduced into fetal MRI image analysis in recent years. In particular, the rapid advancement of deep learning (DL) techniques in medical image processing has greatly facilitated automated segmentation and anomaly detection [6]. Convolutional neural networks (CNNs), owing to their powerful capability in feature extraction and pattern recognition, have been widely used in brain structure segmentation and disease diagnosis [7]. Several studies have applied U-Net and its variants for the segmentation of major fetal brain structures in MRI [8], and further explored their potential in detecting developmental abnormalities [9]. However, the automatic identification of CC abnormalities remains particularly challenging. While the normal CC exhibits relatively consistent morphology, abnormalities typically manifest as alterations in length and thickness [10]. Length changes are relatively easier to assess, whereas accurate measurement of thickness variations presents considerable difficulty due to the structure’s inherently thin profile, limited image resolution, and susceptibility to fetal motion artifacts. Additionally, the small sample size of fetal imaging datasets imposes stringent demands on model robustness and generalization [11, 12].

Existing approaches can be broadly categorized into traditional image-based methods and deep learning-based methods. Traditional imaging techniques mainly rely on morphological features and intensity variations of the CC. After semi-automatic or manual segmentation, structural indices such as thickness and length are measured to assess developmental abnormalities [13]. However, these methods are time-consuming, highly sensitive to image quality, and heavily dependent on manual intervention, thus limiting their clinical applicability. In contrast, deep learning approaches leverage their strong feature-learning capabilities to enable end-to-end automation, reducing operator dependence and improving diagnostic consistency [14]. Recently, CNN-based segmentation architectures such as 3D U-Net and Attention U-Net have achieved promising results in fetal brain MRI segmentation [15–18]. Furthermore, by integrating segmentation networks with anomaly detection or classification models, these approaches have enabled automated identification of developmental disorders [19–21]. Researchers have also explored strategies to optimize performance under limited data scenarios, such as transfer learning, attention mechanisms, and improved loss functions [22–24], in order to enhance model generalization and accuracy.

To address the aforementioned challenges, this study proposes a novel intelligent diagnostic framework for fetal CC based on a “detection-then-classification” paradigm [25]. The proposed framework establishes a fully automated pipeline from raw fetal ultrasound input to abnormality classification output, comprising two key innovations: (1) Accurate CC ROI extraction via YOLOv8: we adopt the state-of-the-art YOLOv8 object detection model to process input ultrasound images [26–29]. This module effectively mitigates the impact of fetal motion and imaging artifacts, enabling rapid and precise localization of the region of interest (ROI) corresponding to the CC [30], thereby providing high-quality input for the subsequent classification stage. (2) Anatomy-aware dual-stream classification network: the extracted CC ROIs from axial and sagittal views are fed into a specially designed dual-stream architecture that processes the two perspectives separately. Through an anatomy-aware feature fusion module [31, 32], the network captures subtle morphological variations indicative of developmental abnormalities, achieving accurate classification results.

All data used in this study were retrospectively collected from the Affiliated Hospital of Jining Medical University, comprising a large number of clinical fetal ultrasound cases for model training and validation. Experimental results demonstrate that the proposed two-stage framework outperforms conventional single-stage methods in detecting CC abnormalities. The main contributions of this work are summarized as follows: (1) A novel two-stage diagnostic paradigm is proposed, improving localization accuracy under complex imaging conditions. (2) The state-of-the-art YOLOv8 model is successfully adapted for precise CC ROI extraction in fetal ultrasound. (3) A dual-stream network integrating multi-view and anatomy-aware representations is designed, significantly enhancing the classification of subtle developmental anomalies.

## 2 Materials and methods

### 2.1 Data collection

This retrospective study aimed to develop and validate an AI-assisted diagnostic framework for fetal corpus callosum abnormality detection using dual-view ultrasound imaging. The study protocol was approved by the Ethics Committee of Jining Medical University. Due to the retrospective nature and use of anonymized imaging data, the requirement for informed consent was waived by the ethics committee. All procedures were conducted in accordance with the Declaration of Helsinki.

The proposed method was validated on two independent fetal ultrasound datasets collected between 2023 and 2025. Dataset I comprised 496 cases from the Department of Ultrasound, Jining Medical University, including 51 cases of CC agenesis and 445 normal cases, used for model development and internal validation. Dataset II included 93 cases (3 agenesis, 90 normal) from Jining Maternal and Child Health Hospital, serving as an independent external test set excluded from model training.

Each case contained high-quality 2D ultrasound images in both sagittal and axial cranial planes, with one diagnostically representative frame selected per view. All data were stored in DICOM format, acquired using Philips EPIQ7 and GE Voluson E10 ultrasound systems. To ensure diagnostic consistency, all images were independently annotated by two senior fetal imaging specialists following international consensus standards, with disagreements resolved by a third expert. Detailed demographic and clinical characteristics are summarized in Table 1.

**Table 1:** Demographic and clinical characteristics of the included datasets.

Variable	Training data	Internal dataset	External dataset
Age (Mean ± SD)	29.68±5.94	29.37±5.87	31.31±6.08
Gestational age (Mean ± SD)	27.53±4.24	27.74±4.44	26.00±4.16
Gravidity (Mean)	2.53	2.47	2.84
Pregnancy complications			
None	276 (79.6%)	384 (77.4%)	85 (91.4%)
Present	71 (20.4%)	112 (22.6%)	8 (8.6%)
CC developmental status			
Normal	311 (90.8%)	445 (89.7%)	90 (96.7%)
Agenesis	36 (9.2%)	51 (10.3%)	3 (3.3%)

### 2.2 Data preprocessing

Considering the imaging characteristics of ultrasound, a three-step preprocessing pipeline was implemented. First, from each video sequence, a single static frame showing the most complete CC morphology was selected for both sagittal and axial views. These two planes were then aligned using affine transformation to ensure spatial consistency across views. Second, adaptive homomorphic filtering was applied to suppress speckle noise and enhance the visibility of low-echo CC boundaries. Finally, both images were uniformly rescaled to 256×256 pixels while preserving the original aspect ratio to minimize deformation caused by probe pressure.

Data partitioning strictly followed patient-level separation. For Dataset I, samples were randomly split into training (70%) and testing (30%) subsets. The training subset was used for model development with data augmentation. Data augmentation strategies included: random rotation within $\pm 15^{\circ }$ to simulate probe angle variation, ±10% brightness adjustment to accommodate acoustic window differences, and elastic deformation (σ=8, α=32) to emulate fetal motion artifacts. To improve prediction robustness and reduce variance during evaluation, we applied test-time augmentation (TTA) to both the internal and external test sets. Specifically, for each test image, we generated five augmented versions using the same augmentation strategies as training (rotation, brightness adjustment, and elastic deformation). It is important to note that the original test images themselves remained unchanged; TTA was applied only during the inference process and did not alter the test datasets. For visualization purposes in Figure 4, we randomly selected a representative subset of these TTA predictions to construct the confusion matrices (500 predictions from the internal test set and 50 from the external test set), while the primary performance metrics reported in the text were computed based on the original test images without TTA to maintain consistency with the case counts in Table 1.

The resulting multimodal dataset comprised paired preprocessed images from axial and sagittal planes, serving as standardized inputs for subsequent ROI localization and classification.

### 2.3 Evaluation metrics

To comprehensively assess the performance of the proposed intelligent diagnostic model for corpus callosum (CC) evaluation, multiple quantitative metrics were employed to measure accuracy and reliability. Specifically, we defined the following evaluation indicators: Accuracy, Recall, Precision, and F1-score (Equations 1–4):

$$\text{Accuracy}=\frac{\text{TP}+\text{TN}}{\text{TP}+\text{TN}+\text{FP}+\text{FN}},$$ (1)

$$\text{Recall}=\frac{\text{TP}}{\text{TP}+\text{FN}},$$ (2)

$$\text{Precision}=\frac{\text{TP}}{\text{TP}+\text{FP}},$$ (3)

$$\text{F1-score}=\frac{2\times \text{Recall}\times \text{Precision}}{\text{Recall}+\text{Precision}}.$$ (4)

where TP and TN denote the numbers of correctly classified positive and negative samples, respectively, while FP and FN represent the incorrectly classified positive and negative samples. In addition, we computed the area under the receiver operating characteristic curve (AUC) to further evaluate classification performance. The closer the AUC value is to 1, the stronger the discriminative ability of the model. Particularly for imbalanced datasets, AUC provides a more stable and unbiased assessment compared to accuracy alone.

For the object detection task, we additionally introduced the Intersection over Union (IoU) metric to evaluate localization accuracy. IoU quantifies the degree of overlap between the predicted and ground-truth bounding boxes, defined as:

$$\text{IoU}=\frac{\text{Area of Overlap}}{\text{Area of Union}}=\frac{\text{Area}(P\cap G)}{\text{Area}(P\cup G)}$$

where P and G denote the predicted and ground-truth bounding boxes, respectively. A higher IoU value indicates more precise localization of the CC and its associated anatomical structures. Through these combined metrics, we were able to comprehensively evaluate model performance in both the detection and classification of normal and absent CC cases, providing a solid basis for further model optimization and refinement.

### 2.4 Overview of the artificial intelligence diagnostic system

**Figure 1:**
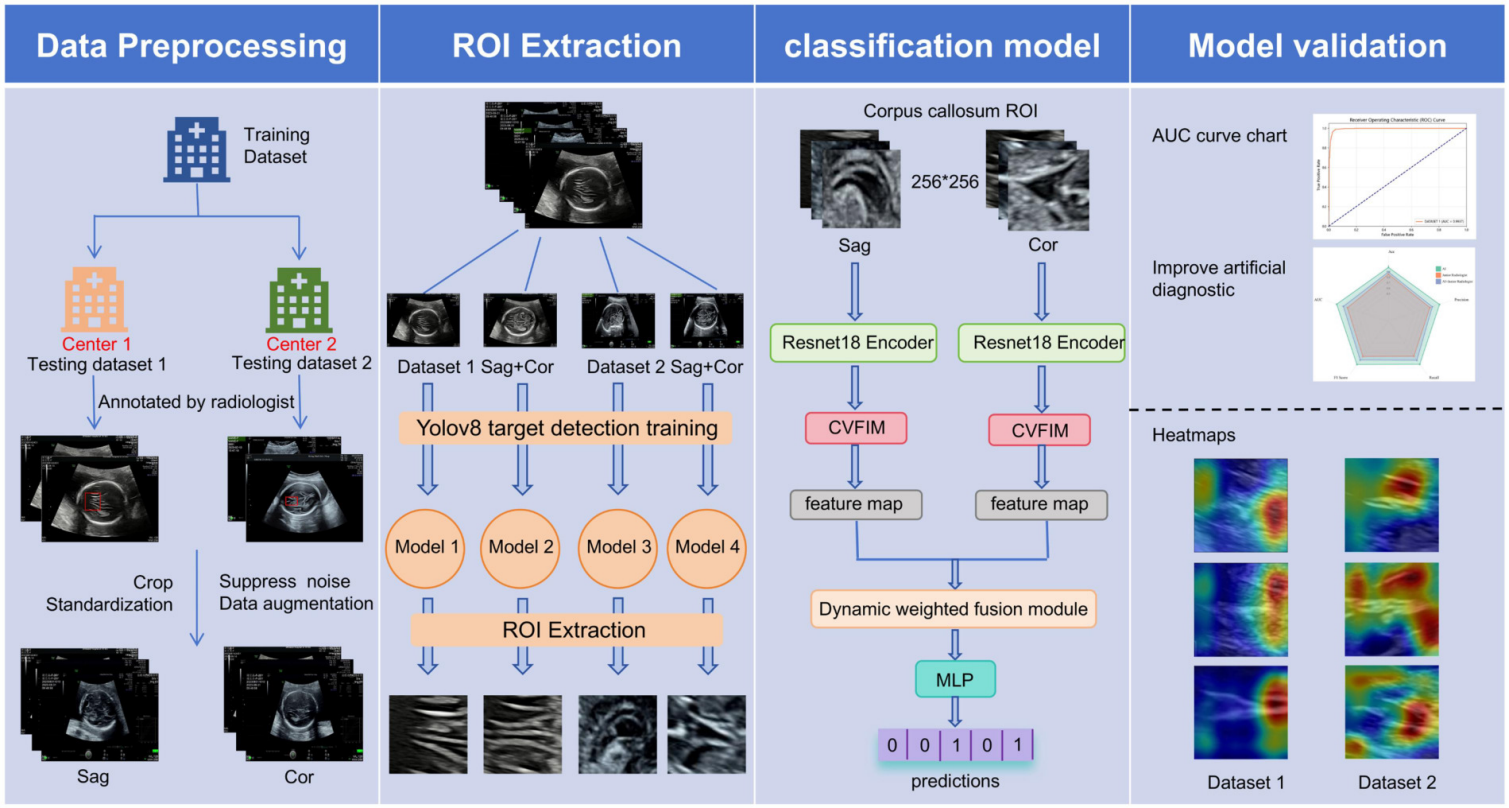
Overall framework of the intelligent diagnostic system.

In this study, we successfully developed an intelligent diagnostic system for the binary classification of normal and absent corpus callosum (CC). As illustrated in Figure 1, the system integrates multiple key modules, including ultrasound data preprocessing, preliminary localization of the CC region, automatic classification of CC status, and model evaluation and visualization.

1. **Accurate ROI Extraction of the Corpus Callosum.** The CC is a relatively small and complex anatomical structure within the fetal brain, exhibiting subtle echogenic characteristics that are easily obscured by surrounding tissues in ultrasound images. To address this challenge, we adopted an advanced target detection framework based on YOLOv8 to precisely localize the CC region, thereby providing high-quality preprocessed data for subsequent analysis.
2. **Lesion Classification with Deep Learning and Expert Interpretation.** By leveraging multi-view ultrasound images, we comprehensively captured the morphological features of the CC. An interactive module was incorporated into the feature extraction network to facilitate feature fusion between different views, providing the classifier with more informative representations. Based on these extracted features, the intelligent classification system can accurately identify normal and absent CCs. Furthermore, visualization techniques such as heatmaps were employed to intuitively display morphological changes, with the highlighted regions showing strong correspondence to areas of clinical concern identified by experienced sonographers. Our proposed approach demonstrated high consistency with clinical diagnoses and can effectively assist physicians in the rapid localization and assessment of CC integrity, thereby supporting more precise clinical decision-making.

### 2.5 Precise ROI extraction of the corpus callosum based on YOLOv8

Accurate localization of the CC is a prerequisite for diagnosing callosal agenesis in fetal cranial ultrasound analysis. Traditional segmentation methods can produce pixel-level masks but are often computationally expensive and sensitive to boundary inaccuracies. To overcome these limitations, we adopted a high-efficiency object detection paradigm instead of complex segmentation procedures, constructing a dedicated CC localization model based on the state-of-the-art YOLOv8 architecture. YOLOv8 integrates a deeply optimized feature pyramid network and an anchor-free detection head, achieving an excellent trade-off between speed and accuracy—particularly suitable for identifying small anatomical targets that occupy a limited proportion of ultrasound images.

To enhance the model’s sensitivity to low-contrast CC structures, we incorporated a multi-scale feature enhancement module into the YOLOv8 backbone. Specifically, an improved Spatial Pyramid Pooling Fast (SPPF) structure was employed to strengthen multi-receptive-field feature fusion. In addition, an adaptive channel compression mechanism was integrated into the neck network to dynamically calibrate channel weights and suppress background noise inherent to ultrasound imaging. These optimizations enabled the model to precisely localize the CC and its clinically associated structures (e.g., anterior horn of the lateral ventricle, cavum septi pellucidi), directly outputting bounding boxes that tightly encapsulate the anatomical region of interest.

Conventional prediction outputs typically restrict bounding boxes to the CC region itself. Considering the inherent uncertainty of ultrasound imaging and clinical diagnostic needs, we introduced a dynamic boundary expansion algorithm. Based on the YOLOv8-generated bounding boxes, this algorithm adaptively enlarges the detection area by 3–5 pixels according to the expected anatomical dimensions and location of the CC in a given imaging plane. This adjustment ensures that the extracted ROI encompasses not only the CC but also adjacent structures such as the ventricular wall and the cavum septi pellucidi margins. These surrounding regions often exhibit indirect signs of callosal agenesis (e.g., ventricular morphology changes) and thus provide critical contextual information for downstream classification.

We trained four YOLOv8 models using sagittal and axial data from internal dataset and external dataset, respectively. The resulting ROIs closely matched the key anatomical regions manually delineated or visually emphasized by clinical experts during diagnosis, encompassing essential features required for clinical decision-making. The proposed method outperformed alternative segmentation-based approaches in both precision and efficiency.

### 2.6 Anatomy-aware dual-stream fusion network for multi-view integration

We proposed an innovative Anatomy-aware Dual-Stream Fusion Network (ADFNet), specifically designed for synergistic analysis of multi-view fetal cranial ultrasound features. The cropped ROI image patches—focused on key anatomical regions and obtained from the prior detection stage—are used as paired inputs (two views per case) to a dual-stream deep convolutional neural network for classification. The core design principle of this architecture lies in the effective fusion of complementary structural information derived from different ultrasound planes (sagittal and axial), enabling a comprehensive assessment of the presence and integrity of the corpus callosum (CC).

#### 2.6.1 Feature extraction

Each stream, corresponding to one imaging view, employs a ResNet-18 backbone pretrained on ImageNet and fine-tuned through transfer learning. The deep residual structure of ResNet-18 facilitates multi-level visual representation learning, while the pretrained weights provide a robust initialization for adapting to CC imaging data. To further enhance inter-view feature interaction, we introduced a lightweight Cross-View Feature Interaction Module (CVFIM) after the third residual stage of the backbone network, enabling intermediate-level information exchange rather than limiting fusion to the final stage. Specifically, the covariance matrix between the two feature spaces is computed to model inter-view correlations, generating complementary attention weights that are added to the original feature maps via a residual-like connection. The formulation is as follows:

$$F_{\mathrm{sag}}^{\prime }=F_{\mathrm{sag}}+\sigma (\phi (F_{\mathrm{cor}}))⊙F_{\mathrm{sag}}$$

where ϕ denotes a 1×1 convolution, σ is the Sigmoid activation, and ⊙ represents element-wise multiplication. This mechanism allows each stream to incorporate contextual cues from the other view before entering the subsequent processing stage, achieving more comprehensive feature fusion.

#### 2.6.2 Feature fusion and classification

At the end of the ResNet-18 backbones, both branches output high-dimensional feature maps. To further improve the integration of these representations, we designed a dynamic weighted fusion module followed by a multi-layer perceptron (MLP) classifier. The features from both streams are first concatenated and passed through a global average pooling (GAP) layer to obtain compact one-dimensional feature vectors preserving essential spatial information. These feature vectors are then processed by the dynamic weighting module, which adaptively learns view-specific contribution weights for each case. The fusion process can be expressed as:

$$F_{\mathrm{fused}}=\alpha ⊙\text{GAP}(F_{\mathrm{sag}})+(1-\alpha )⊙\text{GAP}(F_{\mathrm{cor}})$$

where α represents the learned contribution coefficient of the sagittal view. For instance, in anterior fetal positions, the sagittal view typically dominates (α=0.67±0.08), while in posterior positions, the axial view has greater influence (1−α=0.72±0.06). The fused feature vector is then fed into an MLP decision module consisting of two fully connected (FC) layers. The first FC layer includes 512 neurons with ReLU activation and Dropout (rate=0.5) regularization to enhance generalization. The second FC layer outputs a two-dimensional vector corresponding to the “normal” and “absent” classes, followed by a Softmax activation for probabilistic classification. This architecture ensures that complementary visual information from the two ultrasound views interacts and integrates effectively in the high-level feature space, enabling the network to make diagnostic decisions based on richer and more holistic representations. By transforming multi-view ultrasound analysis from simple feature concatenation to anatomy-guided adaptive decision-making, ADFNet provides a high-accuracy, high-reliability solution for fetal cranial screening, effectively reducing misdiagnosis rates and advancing the precision of prenatal diagnostic technologies.

### 2.7 Implementation details

The proposed framework was implemented using PyTorch and trained on an NVIDIA A600 GPU. For the YOLOv8-based ROI detector, we used the default hyperparameters provided by Ultralytics, with an input size of 640×640 and trained for 100 epochs with early stopping.

For the dual-stream classification network ADFNet, each ResNet-18 stream was initialized with ImageNet pretrained weights. To address class imbalance in the training set (311 normal vs. 36 agenesis), we applied class-weighted cross-entropy loss, where the weight for the agenesis class was set to $w_{\text{agenesis}}=\frac{N_{\text{normal}}}{N_{\text{agenesis}}}\approx 8.64$, and the weight for the normal class was 1.0. The Adam optimizer was used with an initial learning rate of $10^{-3}$, betas=(0.9, 0.999), and a batch size of 32. All input images were resized to 256×256 pixels.

## 3 Results

### 3.1 Corpus callosum region extraction

The corpus callosum (CC) is located at the base of the longitudinal cerebral fissure, in the central region between the two hemispheres. In fetal cranial ultrasound images, the CC typically appears as a slender, hypoechoic (dark) curved structure along the midline of the brain. Relative to the entire cranial plane, which contains multiple anatomical components such as the skull, parenchyma, ventricular system, and choroid plexus, the visible CC region generally occupies less than 5% of the total pixels. This proportion can be even smaller under suboptimal acoustic windows or limited resolution.

The original ultrasound images contain a large amount of irrelevant or even interfering information for CC evaluation, including strong echoes from the skull, background parenchymal texture, anechoic ventricular regions, the choroid plexus, and deep brain structures such as the thalamus and basal ganglia, as well as potential artifacts and noise. Direct classification on whole images would require the model to localize minute, diagnostically relevant regions, which dramatically increases computational cost and training complexity.

To address this, the proposed framework extracts the CC region from the entire cranial ultrasound image. The annotation of both primary and external datasets was independently performed by three experienced sonographers (with 10, 8, and 5 years of prenatal diagnostic experience, respectively) using a professional medical image labeling platform. Each expert annotated the CC and its key neighboring structures (including the cavum septi pellucidi and anterior horns of the lateral ventricles) in both sagittal and axial views. To evaluate inter-observer consistency, 20% of the samples were randomly selected for re-annotation by all experts.

**Figure 2:**
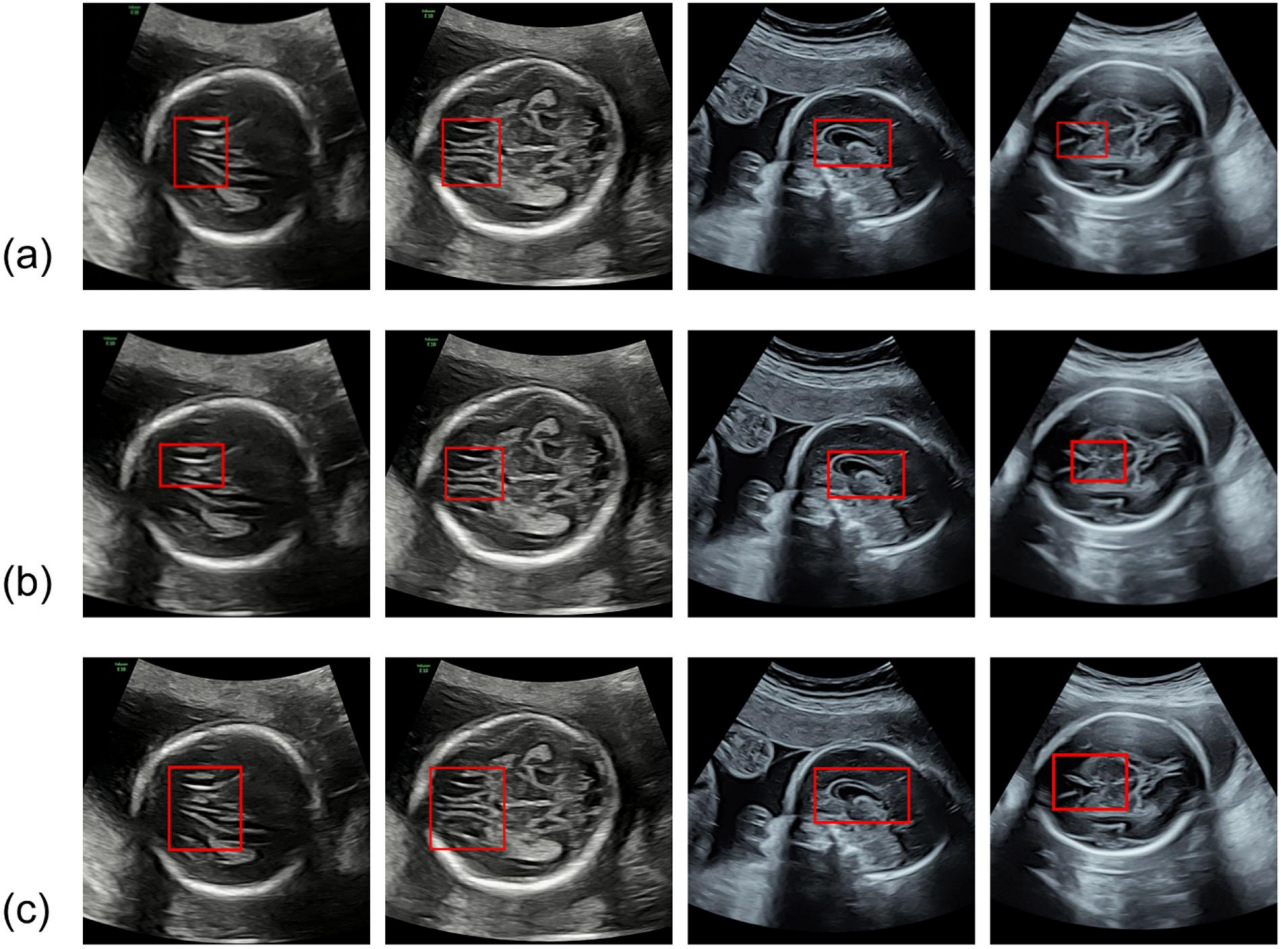
Comparison of corpus callosum ROI extraction methods; **(a)** Proposed method; **(b)** YOLO architecture; **(c)** SSD architecture.

Using a state-of-the-art object detection algorithm, a model was trained to localize the CC and adjacent key anatomical structures. The model takes full-head cranial ultrasound images as input and outputs detection boxes with high confidence. Its localization accuracy, validated through cross-validation, achieves an average Intersection over Union (IoU) of 0.9201±0.030, significantly outperforming the original YOLOv8 (IoU 0.8975±0.041) and SSD (IoU 0.8864±0.038) benchmark models (Figure 2). Compared with manual annotation, the model significantly reduced processing time, saving an average of more than four minutes per image. Based on these detection boxes, regions of interest (ROIs) containing the CC were cropped to form a refined dataset. This strategy focuses the model’s attention and computational resources on the most informative regions, reducing sensitivity to background variation and positional differences while enhancing structural representation learning within the ROI.

### 3.2 Model performance

**Figure 3:**
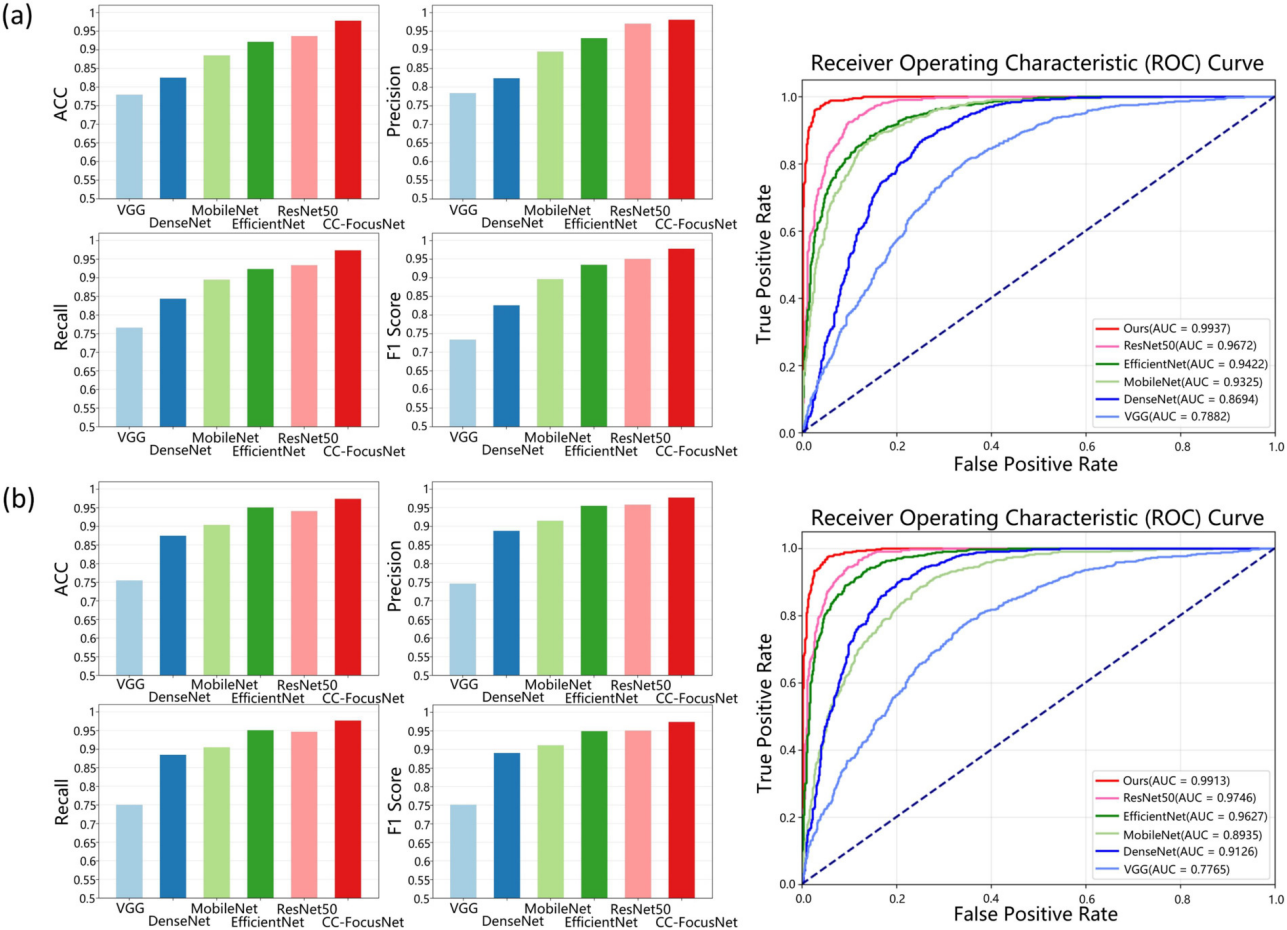
Comparison of CC-FocusNet with other deep learning approaches. **(a)** Comparison results between the proposed method CC-FocusNet and other deep learning methods on internal dataset; **(b)** Comparison results between the proposed method and other deep learning methods on external dataset.

The proposed CC recognition and abnormality detection model was developed using 496 cases from the internal dataset. The inputs consisted of ROI-cropped sagittal and axial ultrasound images, while the external dataset was reserved exclusively for independent testing. The classification performance was evaluated across datasets and compared against alternative deep learning architectures. The quantitative results are presented in Figure 3.

### 3.3 Model performance

**Figure 4:**
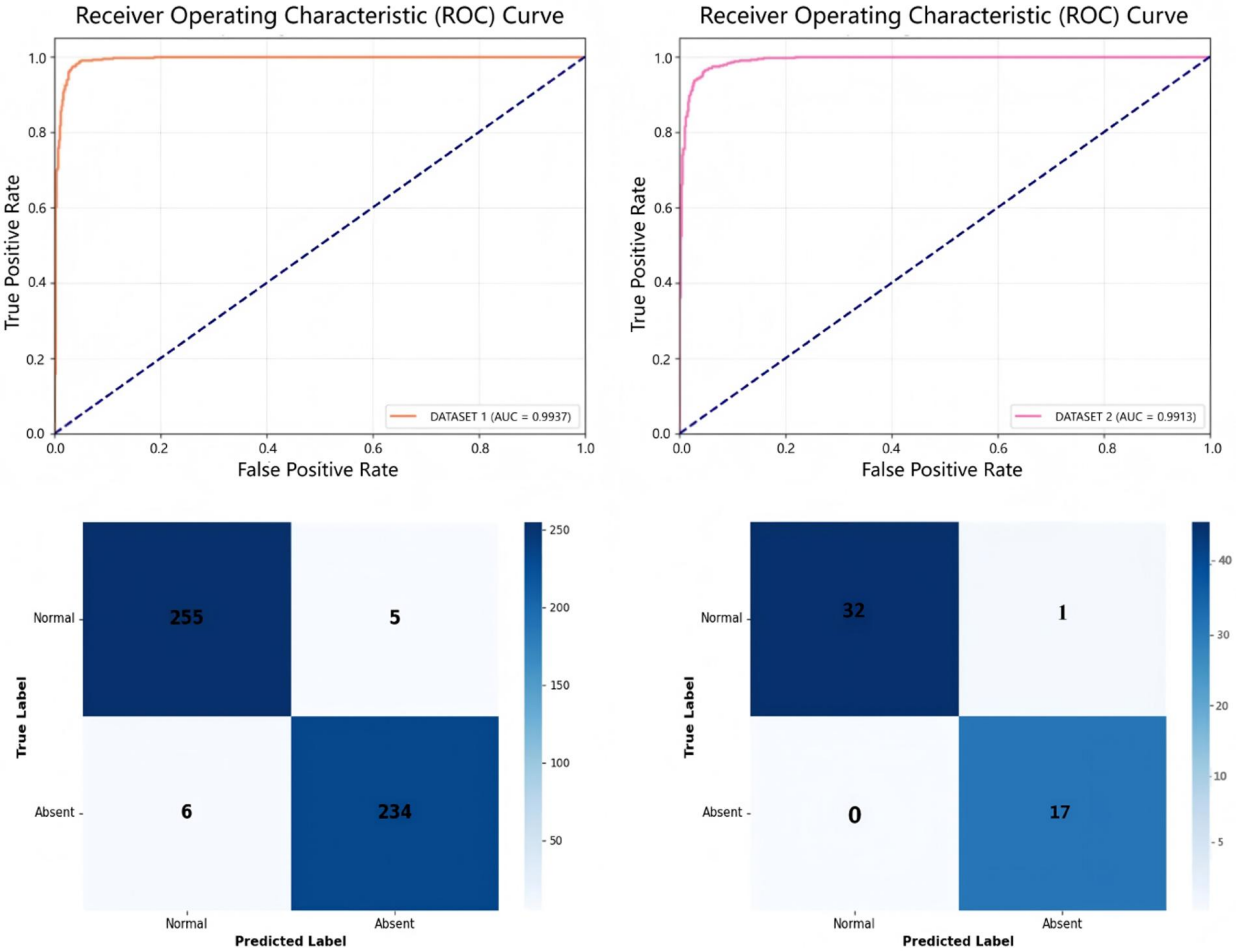
ROC and confusion matrix results for two datasets. (Left) ROC curve and confusion matrix for internal dataset; (Right) ROC curve and confusion matrix for external dataset. Note that the confusion matrices are constructed from a random subset of test-time augmentation (TTA) predictions (500 for internal, 50 for external) for visualization purposes only.

For the internal dataset, our method CC-FocusNet achieved an average accuracy of 97.78%, with precision of 0.9804, recall of 0.9733, F1-score of 0.9776, and an area under the receiver operating characteristic curve (AUC) of 0.9937. When tested on the external dataset, the model achieved an average accuracy of 97.36%, precision of 0.9769, recall of 0.9763, F1-score of 0.9736, and AUC of 0.9913. Our method achieved competitive performance across all metrics relative to the comparative deep learning methods. The AUC comparison indicated no significant difference between internal and external testing, suggesting strong generalizability. The validation ROC curves and confusion matrices for the two datasets are shown in Figure 4. In addition to overall metrics, we computed class-wise sensitivity and specificity to better understand model behavior under imbalance. On the external test set, CC-FocusNet achieved a sensitivity of 97.62% for the agenesis class and a specificity of 96.55% for the normal class, with a balanced accuracy of 97.09%. The internal test set showed sensitivity of 97.50% and specificity of 98.08%, resulting in a balanced accuracy of 97.79%.

By integrating a large-scale, high-quality dataset, anatomy-guided localization with YOLOv8, and a multiscale feature enhancement mechanism, the proposed framework achieved highly accurate classification of CC developmental status. Its diagnostic performance was optimal in standardized data with complete anatomical information and remained robust on an independent external test set, demonstrating strong generalization and clinical applicability under real-world conditions with inherent heterogeneity, noise, and class imbalance.

### 3.4 Model interpretability

**Figure 5:**
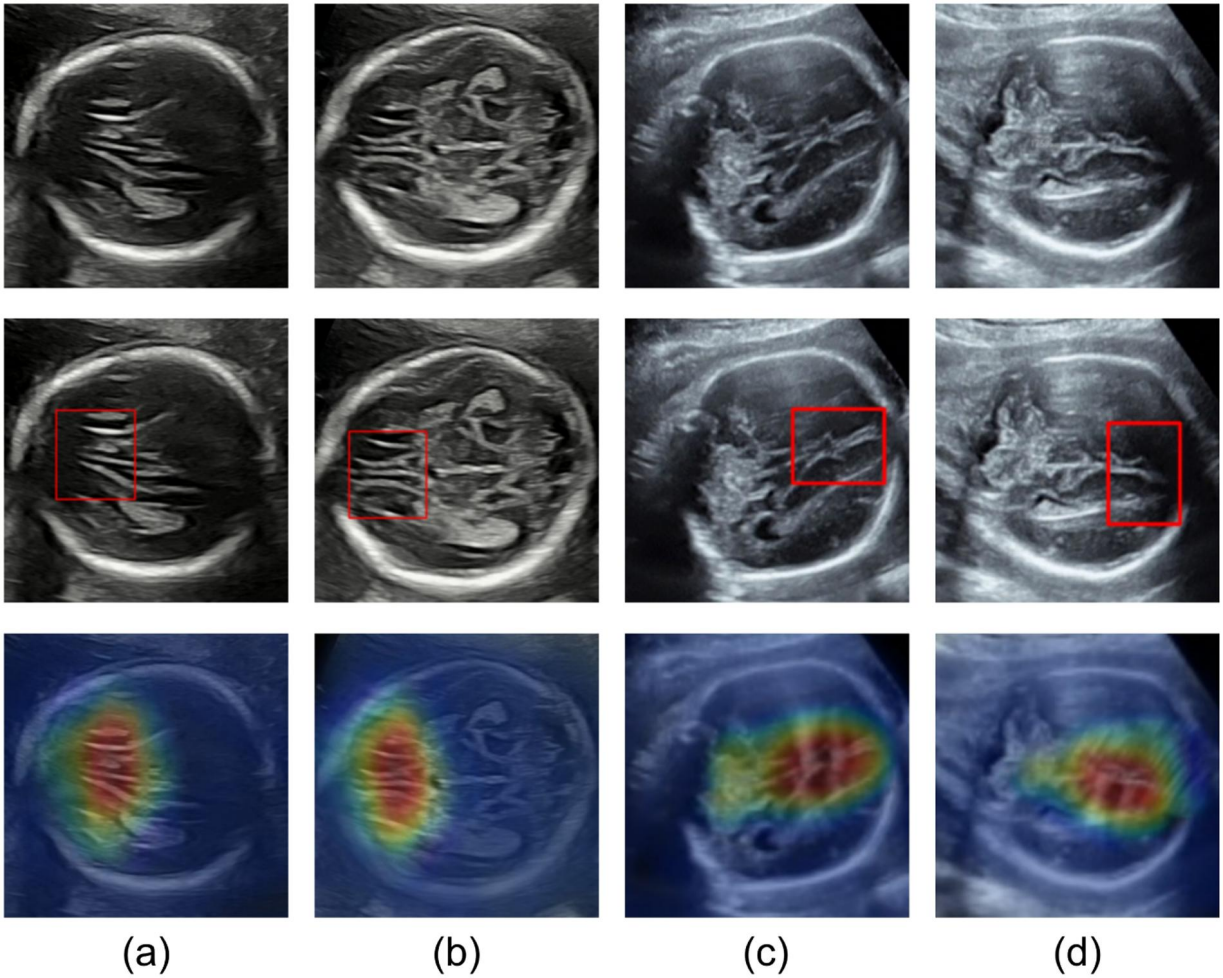
Visualization results for two datasets. **(a)** Model visualization results for internal dataset in the sagittal plane; **(b)** Model visualization results for internal dataset in the axial plane; **(c)** Model visualization results for external dataset in the sagittal plane; **(d)** Model visualization results for external dataset in the axial plane.

To further elucidate the model’s decision-making mechanism, we performed quantitative and visual attribution analysis using Grad-CAM++. The generated activation heatmaps precisely identified the anatomical regions contributing to CC classification. As shown in Figure 5, the heatmaps from correctly classified cases exhibited high spatial concordance with clinically relevant diagnostic regions. The red high-response areas (signal value >200) corresponded primarily to high-echo anatomical landmarks and their surroundings. Since the CC itself is a hypoechoic structure (dark on ultrasound), direct activation is weak; instead, the model infers CC integrity indirectly by focusing on surrounding hyper-echoic cues. In normal cases, high-response regions aligned along the bright falx cerebri dorsal to the CC, representing reinforcement of its bounding landmarks. In agenesis cases, the activation core shifted to the triangular hyper-echoic region formed by the upwardly displaced third ventricle and extended to the bright walls of the dilated lateral ventricles.

The heatmap intensity reflected feature discriminability: deep red regions (signal >220) indicated decisive anatomical evidence (e.g., complete CC interruption), yellow areas (100–200) represented perceptible but less discriminative cues, and blue-green regions (<80) corresponded to background noise. This fine-grained interpretability assists clinicians in identifying key anatomical structures, particularly in subtle cases, and provides valuable references for early diagnosis and intervention of fetal neurodevelopmental abnormalities.

### 3.5 Clinical utility and human–AI collaboration

To evaluate clinical applicability, we randomly selected 100 dual-view cases (50 normal and 50 CC agenesis) and compared diagnostic performance among three groups: the deep learning model alone, junior clinicians (with >3 years of experience), senior clinicians (with >5 years), and a collaborative AI-assisted diagnostic system. For each case, participating clinicians provided both a binary diagnosis (normal or agenesis) and a confidence score ranging from 0% to 100%, indicating their subjective certainty based on the visibility of key anatomical landmarks and overall image quality. The collaboration adopted a dynamic weighted fusion strategy—an “AI-dominant” mode (final score =0.7× AI probability +0.3× clinician confidence) for junior doctors and a “balanced decision” mode (0.5 × AI probability +0.5× clinician confidence) for senior doctors.

**Figure 6:**
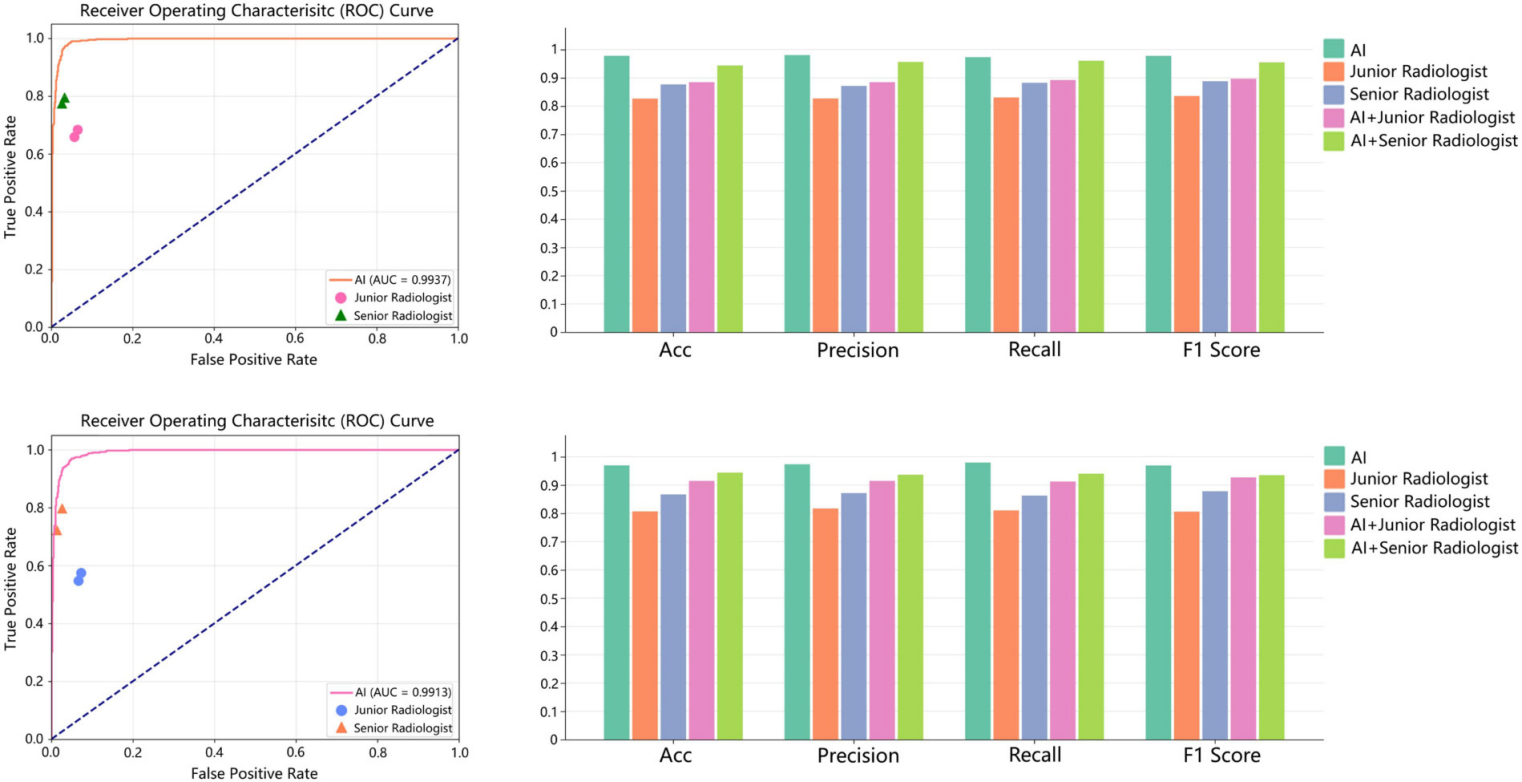
Comparison of AI model, human diagnosis, and collaborative diagnosis results on two datasets (Top: internal dataset; Bottom: external dataset).

As shown in Figure 6, the AI model alone achieved a mean diagnostic accuracy of 97% across datasets, outperforming the junior group (90.1%). The AI–senior collaboration achieved an accuracy of 98.1% on external dataset, improving upon senior clinicians’ independent accuracy (96.8%) by 1.3 percentage points. Notably, the posterior-occipital misdiagnosis rate dropped from 8.7% to 0.9%. The collaborative system also slightly improved recall (98.4% vs. 97.1%) and F1-score (98.7% vs. 97.9%), highlighting the complementary strengths of expert judgment and AI computation. Among seven cases of partial cavum septi pellucidi absence, the collaborative mode successfully avoided all missed diagnoses, whereas the standalone AI model produced one false negative.

In terms of efficiency, the collaboration required an average of 2.1 min per case—longer than AI alone (8.7 s) but 67% faster than senior clinicians working independently (6.5 min), enabling stable throughput of 80 cases per day. These findings demonstrate that our model performs competitively with human experts and that AI-assisted collaboration offers a promising paradigm for prenatal defect prevention, especially in primary healthcare settings where senior imaging specialists are scarce.

## 4 Discussion

Abnormal development of the corpus callosum (CC) is one of the most common congenital malformations of the fetal central nervous system. Accurate identification of CC developmental abnormalities is of great importance for assessing brain development and providing prognostic counseling. Traditional ultrasound screening mainly relies on the operator’s experience in identifying standard planes, and the diagnostic consistency and sensitivity are often limited by operator subjectivity, fetal position, and acoustic window conditions.

To address these clinical challenges, this study proposed a deep learning–based artificial intelligence (AI)–assisted diagnostic framework CC-FocusNet for the automated classification of CC developmental status. The first major innovation of this study lies in overcoming the challenge of precisely identifying small, low-contrast targets in ultrasound images. The CC accounts for less than 5% of the cranial ultrasound field and appears as a hypoechoic structure, which is easily obscured by background noise using conventional methods. By improving the ROI localization module in YOLOv8, our model achieved precise detection of the CC and its associated structures. Notably, the heatmap visualizations demonstrated a high degree of overlap between the model’s decision regions and the clinically relevant areas of interest. This interpretability allows the AI system to serve as a “transparent decision-making partner” for clinicians rather than a black-box tool.

The second major innovation compared with previous studies is that, instead of analyzing a single imaging plane, our framework leverages the anatomical complementarity between the sagittal and axial views. Through dynamic, adaptive weighting, the system balances the contribution of each view to address the core challenge of fetal positional variability in ultrasound imaging. Specifically, the sagittal plane is critical for assessing the relationship between the CC and the cavum septi pellucidi complex. When sagittal imaging is affected by acoustic shadows, the axial plane—less hindered by cranial bone interference—can provide clearer visualization of the ventricular structures and verify suspected abnormalities. This strategy not only enhances the model’s robustness in complex clinical settings but also establishes a new paradigm for multimodal and multiplanar ultrasound intelligence analysis.

Furthermore, we constructed a multicenter fetal cranial ultrasound dataset to comprehensively evaluate deep learning–based imaging models for CC abnormality classification. Our model provides a benchmark for this dataset, laying the groundwork for broader applications in prenatal screening and diagnosis. The experimental results demonstrated superior diagnostic performance across two independent datasets, confirming the clinical translation potential of AI in fetal brain malformation screening.

However, several limitations should be acknowledged. The dataset contained relatively few cases of CC agenesis and no cases of hypoplasia, reflecting the real-world imbalance in disease prevalence but potentially reducing model sensitivity to rare cases. Furthermore, the external test set contained only three cases of CC agenesis, which restricts the robustness of generalization estimates. This limited sample size for the abnormal class reduces the statistical power of our findings on that subset. Although multicenter data were used, further optimization of ultrasound acquisition parameter standardization is needed. Moreover, despite the use of data augmentation, the limited number of abnormal cases remains a challenge. Future work should focus on integrating larger-scale, multicenter imaging data under privacy-preserving frameworks and incorporating synthetic oversampling methods such as SMOTE to enhance the model’s ability to detect rare abnormalities, thereby improving model generalizability. In addition, the current model is based on 2D sectional imaging and does not fully exploit the spatial anatomical information available in three-dimensional ultrasound or 3D reconstruction techniques. Future studies could explore 3D volumetric data to enable comprehensive structural evaluation of the CC, advancing fetal brain development assessment from morphological diagnosis to precise quantitative analysis.

## Data Availability

The data analyzed in this study is subject to the following licenses/restrictions: The dataset contain sensitive medical information. Due to patient privacy regulations, institutional policies, and ethical approval conditions, the data cannot be shared publicly. Requests to access these datasets should be directed to xuxuanjyfy@163.com.
